# A Cohort Study of Lymphatic Filariasis on Socio Economic Conditions in Andhra Pradesh, India

**DOI:** 10.1371/journal.pone.0033779

**Published:** 2012-03-19

**Authors:** Suryanaryana Murty Upadhyayula, Srinivasa Rao Mutheneni, Madhusudhan Rao Kadiri, Sriram Kumaraswamy, Balakrishna Nagalla

**Affiliations:** 1 Bioinformatics Group, Biology Division, Indian Institute of Chemical Technology (CSIR), Hyderabad, Andhra Pradesh, India; 2 National Institute of Nutrition (ICMR), Hyderabad, Andhra Pradesh, India; Kenya Medical Research Institute - Wellcome Trust Research Programme, Kenya

## Abstract

**Background:**

To assess the impact of socioeconomic variables on lymphatic filariasis in endemic villages of Karimnagar district, Andhra Pradesh, India.

**Methods:**

A pilot scale study was conducted in 30 villages of Karimnagar district from 2004 to 2007. These villages were selected based on previous reports from department of health, Government of Andhra Pradesh, epidemiology, entomology and socioeconomic survey was conducted as per protocol. Collected data were analysed statistically by Chi square test, Principal Component Analysis, Odds ratio, Bivariate, multivariate logistic regression analysis.

**Results:**

Total of 5,394 blood samples collected and screened for microfilaria, out of which 199 were found to be positive (3.7%). The socioeconomic data of these respondents/participants were correlated with MF prevalence. The socioeconomic variables like educational status (Odds Ratio (OR) = 2.6, 95% Confidence Interval (CI) = 1.1–6.5), house structure (hut OR = 1.9, 95% CI = 1.2–3.1; tiled OR = 1.3, 95% CI = 0.8–2) and participation in mass drug administration program (OR = 1.8, 95% CI = 1.3–2.6) were found to be highly associated with the occurrence of filarial disease. The socioeconomic index was categorized into low (3.6%; OR-1.1, 95% CI: 0.7–1.5) medium (4.9%; OR-1.5, 95% CI = 1–2.1) and high (3.3%) in relation to percentage of filarial parasite prevalence. A significant difference was observed among these three groups while comparing the number of cases of filaria with the type of socioeconomic conditions of the respondents (P = 0.067).

**Conclusions:**

From this study it is inferred that age, education of family, type of house structure and awareness about the filarial disease directly influenced the disease prevalence. Beside annual mass drug administration program, such type of analysis should be undertaken by health officials to target a few socioeconomic factors to reduce the disease burden. Health education campaigns in the endemic villages and imparting of protection measures against mosquitoes using insecticide treated bed nets would substantially reduce the disease in these villages.

## Introduction

Lymphatic filariasis (LF), the second most common vector-borne parasitic disease after malaria, is found in 81 tropical and subtropical countries [1], [2]. World Health Organisation (WHO) estimates that 120 million people are infected with this parasite and 1.3 billion (i.e. >20% of the global population) are living at risk of infection. It is estimated that 40 million people are suffering from the long term complications of the disease [3]. One-third of people infected with LF live in India, one third live in Africa and the remainder live in the Americas, the Pacific Islands, Papua New Guinea and South-East Asia [4]. The Global Programme for Elimination of Lymphatic Filariasis (GPELF) began its campaign to interrupt transmission of the parasite using a strategy of annual mass drug administration (MDA) to those at risk and to control or prevent LF-related disability through morbidity management programs in which 12 million people have been treated Since 2000 [5]. The latest WHO figures shows that around 381 million people received filariasis treatment in 2005 alone in 42 countries [6]. In India LF is endemic in 18 states and the Union Territories. Approximately 420 million people reside in endemic areas and 48.11 million are infected. Mortality is uncommon, whereas morbidity associated with this infection can be considerable and lifelong. Because of these factors, LF escapes the attention of planners and governments. Rural and urban areas in India suffer with lack of adequate antifilarial measures and it is estimated only 11% of the endemic population is protected by the National Filaria Control Programme (NFCP), Government of India [7].

LF causes a wide spectrum of clinical manifestations in the infected populace. Most of the population suffer with symptoms of LF such as chronic lymphoedema, elephantiasis and hydrocele. Those infected with LF further bear the debilitating effect of acute filarial attacks that last from five to seven days and may occur two to three times each year. Chronic filarial disease has serious social and economic effects. Those afflicted with elephantiasis and hydrocele are often socially marginalized and poor. Acute attacks and chronic disability cut economic output and increase poverty [2], [8]. This is evident from the observation that 94% of the countries with the lowest human development index (HDI) are endemic for LF [9]. The chronic manifestations of filariasis can have significant, and often very negative, social impact [10]. LF has traditionally been considered to be a disease associated with poverty, inadequate sanitation and underdevelopment [9], [11], [12], [13], [14]. Sociodemographic factors such as ethnic group, parent's education and occupation, use of protective measures, and living standard of the family are suggested to be important risk factors for epidemics of vector borne disease [15]. From filarial endemic countries there is little published evidence of an association between LF and country-level poverty [16]. In Philippines, there is an apparent association between LF endemicity and poverty at provincial level [11]. In the majority of control strategies, the target population of disease transmission and control are overlooked. In filariasis, poor knowledge and indigenous, traditional belief systems contribute to high-risk and inappropriate illness prevention and treatment [17].

Lymphatic filariasis (LF), caused by *Wuchereria bancrofti* and transmitted by the Southern house mosquito *Culex quinquefasciatus*, accounts for 95% of the total LF cases in India [18]. To asses the LF disease and its biased factors, a pilot scale study was carried out in Karimnagar district of Andhra Pradesh. The villages of this district have been recognised as endemic for filariasis and MDA programs are still going on. There are no such reports available on impact of socio-economic factors on LF in Andhra Pradesh. Hence, the aim of this study is to assess the relationship between socioeconomic status and occurrence of LF in these villages of Karimnagar district of Andhra Pradesh.

## Results

### Parasitological survey

**Figure 1:**
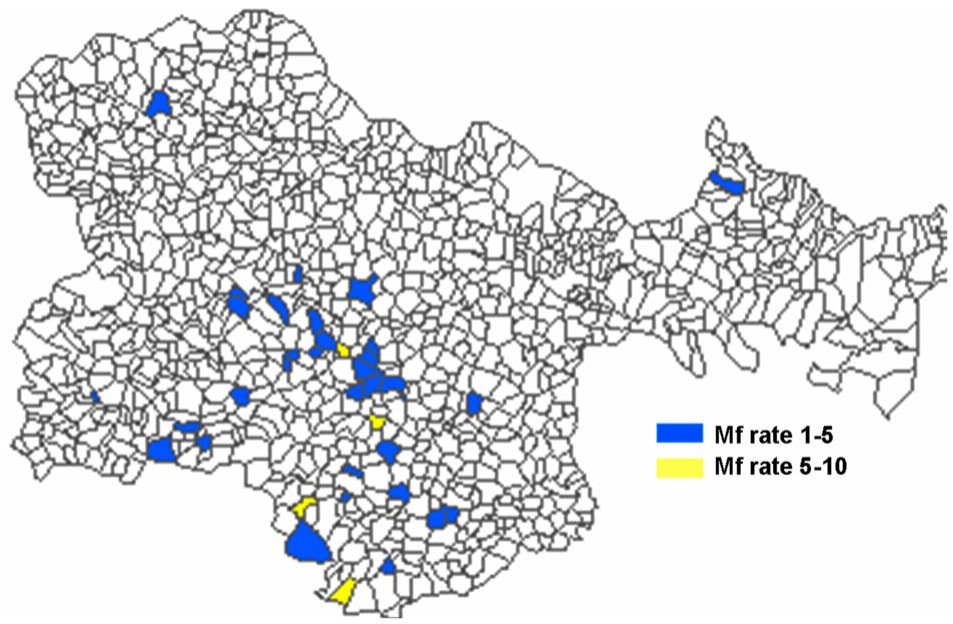
Distribution of microfilaremia prevalence in villages of Karimnagar district of Andhra Pradesh, India.

The magnitude of microfilaremia prevalence rate in the study area ranged from 0 to 10.5% (Figure 1). During filarial survey 5,394 blood samples were collected from 30 villages, out of which 2,771 (51.41%) were females and 2,623 (48.68%) were males. Among 5,394 blood samples, 199 of them were found to be positive for microfilaria (3.7%).

### Association of Filaria with socioeconomic indicators

Among 199 samples, while comparing the MF rates between male (3.9%) and female (3.5%), there is no significant differences observed in the positive cases (P = 0.448). Hence it is inferred that, MF infection is not gender specific. However, a correlation is observed between the various age groups of respondents/participants and percentage on number of filarial cases. The filarial cases were found to increase significantly in number with the increase in age groups (P = <0.001). In these study areas most of the respondents practice agriculture as their key occupation (40.6%) followed by labourers (39.6%). While comparing the filarial scenario with type of occupation, the microfilaria positive case significantly differed with the type of occupation (P = 0.049). Data on monthly income was collected from the respondents. It was found that majority of respondents monthly income ranged from Rs.1,000 to 3,000 (62.7%). While comparing the number of positive cases with monthly income, a significant high number of filarial cases were observed among the low income group of respondents (<1,000/month) (P = 0.020). In most of the Indian villages, people live in lowly constructed house types. In this study, it is noticed that, 44% live in tiled and 28.8% in hut type of houses. While comparing the type of housing structure with the number of positive cases significant differences were observed among hut, tiled and reinforced cement concrete (RCC) (P = 0.032). In the surveyed villages, most of the populace are illiterates and mostly undergraduates (93.9%). High numbers of microfilaria positive cases were recorded in undergraduate (3.8%) when compared with graduates (2.4%) (P = 0.219) (Table 1).

**Table 1:** Filaria prevalence, socioeconomic status and socioeconomic indexes used for principal component analysis in Karimnagar district of Andhra Pradesh.

Variables	Categories	Survey Samples (%) (n = 5394)	Microfilaria Parasite prevalence (%)	χ^2^	P – Value
Age	1–5	3.2	0	91.95	<0.001
	6–10	10.2	0.9		
	11–17	19.2	1.4		
	18–25	15.4	2		
	26–40	26.5	4.4		
	41–60	22.4	7.1		
	>61	2.9	8.2		
Gender	Male	48.6	3.9	0.58	0.448
	Female	51.4	3.5		
Occupation	Agriculture	40.6	4.2	9.537	0.049
	Labourers	39.6	3.2		
	Business	10.9	3.4		
	Employees	3.4	1.1		
	Others	5.5	5.7		
Education	Undergraduate	93.9	3.8	1.513	0.219
	Graduate	6.1	2.4		
Income (INR/Rs.)	<1000	23.2	4.9	7.782	0.02
	1000–3000	62.7	3.5		
	>3000	14.1	2.6		
House structure	Hut	28.8	4.2	6.896	0.032
	Tiled	44	4		
	R.C.C	27.2	2.6		
Breeding Habitats	Cess Pool	18.4	3.5	14.516	0.002
	Cess Pit	14.5	3.1		
	Open drainage	36.2	4.9		
	No, breeding habitats	31	2.6		
Drainage system	Kutcha	25.2	5.6	11.975	0.001
	Pucca	74.8	3.4		
Mosquito avoidance	Yes	18.2	4.1	0.531	0.466
	No	81.8	3.6		
Participated in MDA program	Yes	58.3	4.5	13.422	<0.001
	No	41.7	2.6		
Filaria awareness	Yes	71	3.8	0.175	0.676
	No	29	3.5		

As mentioned earlier, the present study area comes under filariasis endemic region and MDA program is still continuing in these regions (P = <0.001). So, when compared to other districts, the population of Karimnagar have good knowledge about this disease. The collected data shows that nearly 71% of population are well aware about filariasis. While comparing the filarial awareness among the respondents, it is noticed that higher percentage of MF was recorded among the disease unaware respondents than the aware respondents. Although the percentage difference between aware (3.8%) and unaware respondent (3.5%) was extremely low, and not statistically significant (P = 0.676). In this quantitative survey, mosquito protection measures were also analyzed. It is noticed that, a lower proportion of population use the mosquito protection measures such as bed nets, coils and other mosquito repellents (18.2%), whereas 81.8% of population do not use any such precautionary measures. While comparing these two groups it was found that they were not statistically significant to the incidence of filariasis (P = 0.466). Data on drainage system of the survey area showed that, majority of area had pucca drainage (74.8%) and kutcha drainage systems (25.2%). A significant increase in the number of positive cases were recorded from pucca drainage than the kutcha drainage (P = 0.001) (Table 1). During the survey it was also observed that there were plenty of mosquito breeding habitats in and around the villages such as cess pools, cess pits and open drainages with stagnated water. The number of filarial positive cases was found to be more in the open drainages where mosquitoes breed and transmit the disease.

Socioeconomic details and prevalence of filariasis variables were examined to determine the influence of socioeconomic variables on filariasis by bi and multi variable logistic regression analysis. The results are shown in table 2. Most importantly, income was found to predict adherence. The income grades are in decreasing order of strength of association with <1,000 (Odds Ratio (OR) = 1.9, 95% confidence interval (CI) = 1.1–3.2) and 1000–3000 (OR = 1.3, 95% CI = 0.8–2.2). In bi variate analysis, lower age group (< = 25) considered as a reference and the analysis reveals that lower age group had significantly lower risk, and risk of filaria increases with increase of age viz; 26–40 (OR = 3.3, 95% CI = 2.1–4.9), 41–60 (OR = 5.4, 95% CI = 3.7–8.1) and > = 61 (OR = 6.3, 95% CI = 3.3–12.2). Multivariable modeling was then performed to refine a bivariate model. A statistical significance was observed in the age groups, education, house structure, drainage system and participation in MDA program was considered for multivariate analysis. Gender, occupation, income, breeding habitats, mosquito avoidance and filarial awareness did not influence the model and has not been considered. The predictors like age groups, educational status (OR = 2.6, 95% CI = 1.1–6.5), house structure (hut OR = 1.9, 95% CI = 1.2–3.1; tiled OR = 1.3, 95% CI = 0.8–2) and participation in MDA program (OR = 0.5, 95% CI = 0.4–0.8) influenced filariasis.

**Table 2:** Bivariable and multi variable analyses of predictors of observance to a filaria cases and socioeconomic survey participants from villages of Karimnagar district of Andhra Pradesh state.

Variables	Categories	Bivariate analysis	Multivariate analysis
		OR (95% CI)	OR (95% CI)
Age	< = 25	Reference	Reference
	26–40*	3.3 (2.1–4.9)	3.6* (2.3–5.6)
	41–60*	5.4 (3.7–8.1)	5.9* (3.8–8.9)
	> = 61*	6.3 (3.3–12.2)	7.3* (3.6–14.6)
Gender	M	1.1 (0.8–1.5)	---
	F	Reference	---
Occupation	Agriculture*	3.9 (1.0–16.1)	---
	Labourers	3.0 (0.7–12.4)	---
	Business	3.2 (0.7–13.8)	---
	Others*	5.5 (1.2–23.9)	---
	Employee	Reference	---
Education	Undergraduates	1.6 (0.8–3.2)	2.6* (1.1–6.5)
	Graduate	Reference	Reference
Income	<1000	1.9* (1.1–3.2)	---
	1000–3000	1.3 (0.8–2.2)	---
	>3000	Reference	---
House Structure	Hut	1.6* (1.1–2.5)	1.9* (1.2–3.1)
	Tiled	1.6 *(1.1–2.3)	1.3 (0.8–2)
	RCC	Reference	Reference
Breeding habitats	Cess pit	0.9 (0.5–1.5)	---
	Cess pool	Reference	---
	No breeding habitats	0.7 (0.5–1.2)	---
Drainage system	Open drainage	1.4* (1.0–2.1)	---
	Kutcha	1.7* (1.3–2.3)	1.5* (1.1–2.1)
	Pucca	Reference	Reference
Mosquito Avoidance	Yes	Reference	---
	No	0.9 (0.7–1.3)	---
Participated in MDA program	Yes	Reference	Reference
	No	0.6* (0.4–0.8)	0.5* (0.4–0.8)
Filariasis awareness	Yes	Reference	---
	No	0.9 (0.7–1.3)	---

* p<0.05.

All socioeconomic factors (Occupation, Education, Income, House Structure, Breeding habitats, Drainage System, Mosquito avoidance, Filaria awareness and Participation in MDA program) with relevant contributions to the combined socioeconomic score were used to generate a combined socioeconomic index by PCA. The results of the PCA are presented in tables 3 & 4. The eigenvalues demonstrate that three factors (factors-1, 2 & 3 in table 3) had weightage >1 (2.14, 1.81 & 1.08) and thus was suited to appropriately represent the socioeconomic status in further analyses. The factor-1 consists of five socioeconomic variables like occupation, income, house structure, filaria awareness and participation in MDA program with 23.85% of variance. Factor-2 comprises of two variables such as drainage system and breeding habitats of mosquitoes with 20.15% of variance. Similarly, factor-3 had education as main variable with 12.03% of variance. The cumulative variance of the three factors is 56.04% (Table 3). The factor loadings have been obtained by using varimax rotation component matrix (Table 4). Using the weights from the principal component, a value for each socioeconomic factor was obtained, which increased with increasing socioeconomic conditions. Based on the per cent rank derived from these values, the socioeconomic status/index was classified into three groups as low, medium and high respectively. Using logistic regression, the odds ratio for filaria prevalence for each of the three socioeconomic categories were calculated. The proportion of socioeconomic status/index for filaria prevalence was 3.6%, 4.9% and 3.3% among the low, medium and high socioeconomic groups (P = 0.067). The odds ratio to the socioeconomic status when compared with the reference group (high) exhibited that the filaria was high in middle socioeconomic group (OR-1.5, 95% CI = 1–2.1, P = 0.02) than the low socioeconomic index (OR-1.1, 95% CI: 0.7–1.5) (Table 5).

**Table 3:** Eigenvalues for principal component analysis (PCA).

Factor loadings	Eigenvalues		
	Total	% of Variance	Cumulative %
Factor-1	2.147	23.855	23.855
Factor-2	1.814	20.158	44.013
Factor-3	1.083	12.033	56.045
Factor-4	0.924	10.262	66.307
Factor-5	0.835	9.281	75.589
Factor-6	0.729	8.104	83.693
Factor-7	0.604	6.711	90.404
Factor-8	0.444	4.929	95.333
Factor-9	0.42	4.667	100

**Table 4:** Varimax rotation component matrix of principal component analysis.

Observed variables	Factor loadings		
	1	2	3
Occupation	−0.587	−0.003	0.027
Education	−0.09	−0.017	0.909
Income	0.581	0.445	0.429
House Structure	0.565	−0.116	0.21
Breeding habitats	0.118	−0.771	0.058
Drainage System	0.112	0.845	−0.025
Mosquito avoidance	−0.37	0.39	−0.444
Filaria awareness	0.637	0.184	−0.065
Participated in MDA program	0.618	−0.342	−0.019

**Table 5:** Microfilaria prevalence according to socioeconomic index (developed using all socioeconomic variables) in Karimnagar district of Andhra Pradesh.

Variables	Categories	Survey Samples (%)	Parasite prevalence (%)	χ^2^	P – Value	Bivariate analysis OR (95% CI)
Socioeconomicindex	Low	34.24	3.6	5.4	0.067	1.1 (0.7–1.5)
	Medium	32.83	4.9			1.5* (1.1–2.1)
	High	32.93	3.3			Reference

* p<0.05.

## Discussion

LF is considered to be one of the principal neglected diseases [19] because of its wide geographic distribution especially in the rural areas. Victims of this disease mostly are poor who live in favourable conditions for the mosquitoes to transmit the disease easily. There are several reports available on various influencing factors for LF incidences and also many workers stated the consequences of socio-economic burdens due to LF [20], [21], [22]. But in this study we have tried to understand how such socioeconomic conditions of rural people would be able to influence the disease burden and what factors mostly regulate the LF intensity in Karimnagar district of Andhra Pradesh. As the disease rate is alarming in this district, India's National Vector Borne Disease Control Programme (NVBDCP) has scaled up MDA to interrupt LF transmission over the past several years and provides diethylcarbamazine (DEC) on a regular interval as a mass drug treatment in these localities [23].

The result from the epidemiology survey reveals that lymphatic filariasis is still prevalent in the Karimnagar district of Andhra Pradesh, India. Although hypo endemic microfilaria (MF) rates were observed in many villages which may be due to the impact of MDA program since 2004 but in some places, the intensity is high which may be due to non-participation in the MDA program (hyper endemic - Mannempalli village, 10.5%; mesoendemic - Basavapuram 6.5%, Ramavaram 5.5% and Parlapalli 5% villages). This result clearly indicates that the availability of microfilaria is still prevalent in these villages. The MF infection pattern among male and female respondents was not statistically significant and the infection was almost equal, which may be due to similar nature of work carried out by males and females. Similar kinds of reports were also noticed in South East Nigeria [24]. As the study area belongs to rural areas where both male and female respondents generally depend on working in agricultural fields (40.6%) and 39.6% as labours. Hence there is high chance of exposure to the similar load of pathogen among male and female. The MF rate among the respondents was found to be increased with the different age groups. In the present study, it is clearly demonstrated that, the Mf rates increased with rise in age groups (1–5∶0%; 6–10∶0.9%; 11–17∶1.4%; 18–25∶2%; 26–40∶4.4%, 41–60∶7.1% and >61∶8.2). Similar kind of results was also reported by Stolk *et al*. (2004) [25]. From the study high MF rates were recorded in adults and older persons than children. These types of reports were also observed in other parts of Andhra Pradesh [26].

Endemicity of LF depends on the population, living conditions and environmental sanitation, socioeconomic and demographic factors are implicated in controlling LF in rural area in Kenya [27], [28]. In the present study occupation and income were found to be significant with the microfilaria prevalence. The occupation of the inhabitants was mainly agriculture, labourers followed by people pursuing their family vocations. It was found that the disease prevalence was significant among those living in close proximity to irrigated agricultures and labourers (engaged in agricultural practices). Agriculture can facilitate the proliferation of mosquitoes including those transmitting filaria [29]. However, in the study area most of the population are low (<1,000) and middle income group (1,000–3,000) house holds and are more risk prone to filariasis. High and middle income participants are generally benefited from clean homes and facilities to maintain personal hygiene and they could afford the cost of the treatment for filariasis. Low income participants lived in less-hygienic conditions and thus were more prone to the infection. Earlier studies reported that low income people are more at risk to lymphatic filariasis and the disease burden is relatively higher in this group of population [30].

The type of housing structure plays an important factor for the abundance of *Cx. quinquefasciatus*. Higher densities of mosquitoes were generally found in homes poorly constructed than the well constructed house [31]. It was also reported that, the density of *Cx. quinquefasciatus* and transmission of filaria is highly correlated with the type of house construction standards [32]. In the present study, there is significantly higher number of MF rates related in Hut/thatched and tiled houses than in RCC constructed houses. The study made evident that construction of houses play an important role in the vectors resting preferences (poor ventilation, walls are made up of mud, opportunities for availing darker places, increased percentage of carbon dioxide due to more persons inside the house, controlled temperature and humidity) as well as density, in poorly constructed houses. It thereby increases the possibility of filarial infection inside houses and thus maintaining a higher potential for filarial transmission among these residents.

Bancroftian filariasis is prevalent both in urban and rural areas and the parasite is transmitted by the tropical house mosquito, *Cx. quinquefasciatus* the main vector for lymphatic filariasis in India [18]. These vectors breed where there is lack of basic sanitary conditions such as in cesspit and kutcha drains [27], [33]. In the study area, it is also observed that most of the villages are with poor drainage systems, sediment with solid wastes, the sewage disposal system was transformed into rudimentary cesspits, ditches that might have significantly favoured for proliferation of *Cx. qninquefaciatus*. Beside these, there are also several breeding habitats like cess pool, open and kutcha drainage that has become ideal breeding grounds for this vector.

Higher percent of filariasis positive cases were noticed among the illiterate/undergraduate than the graduate respondents, however there is no statistical significant difference observed between these two categories (P = 0.219). Similar types of results were obtained by Muhondwa (1983) [34] and Lu *et al.* (1988) [35]. Data on awareness on LF shows that, <15% of the people are aware of the mosquitoes role in the transmission of filariasis in different countries [34], [35], [36], [37]. In this study it is noticed that, nearly 71% of respondents are aware of lymphatic filariasis although nearly 93.9% of respondents are illiterate/below under graduate. Higher percentage of awareness about filariasis among these villagers may be due to the frequent visits of health officials, conducting disease surveillance and implementation of MDA programs. From this data it is inferred that, prevalence of disease is not directly influencing on the awareness/un awareness about the disease. Beside awareness and education, the most important factor is the practice of personal protection measures towards mosquitoes which have direct impact on the disease prevalence. A significant association between not using a mosquito net and presence of microfilaremia was reported by De Albuquerque *et al.* (1995) [38]. In this study also it is noticed that majority of the respondents are aware about the disease transmitted by mosquitoes but they are not implementing personal protection measures due high recurring cost and most of the respondents feel that allergy, breathing problems, cough and head ache could be due to the mosquito repellents [39]. During the survey high prevalence of filariasis (4.5%) is found in respondents participating in MDA program than the non respondents (2.6%). This may be due to DEC not being consumed due to adverse effects of the drug. It also suggests that in this study area the low literacy rate of the respondents plays a big role on the individual's ability to comprehend the necessity of preventive care utilization [40].

In Andhra Pradesh about 16 districts are reported endemic for LF though MDA program in continuing since 2004 [41]. In the study areas 58.3% of population are participating in the MDA program. Out of 30 surveyed villages, six villages have reported ‘0’ microfilaria rate and in these villages inhabitants are aware about the disease and consuming the drugs effectively, in rest of the villages people's knowledge about transmission and prevention of filariasis and mosquitoes is very poor. PCA a statistical technique for selecting the socioeconomic indicators associated with the risk of transmitting lymphatic filariasis and for identifying socioeconomic conditions at risk, such that most of the microfilaremia cases are situated in the low (OR = 1.1, 95% CI = 0.7–1.5) and medium (OR = 1.5, 95% CI = 1.1–2.1) risk section.

From this study it indicates that low and medium socioeconomic conditions and disease prevalence rates favour the probability of LF in the study area. The results from this study make it possible to recognize that areas with similar socioeconomic characteristics had different prevalence rates which are influenced by factors which need to be considered, such as the proximity of water sources and migration [42]. In areas where no cases of filarial infection have been identified but suitable environmental conditions for disease transmission exist, a territorially based surveillance system needs to be created to detect new foci of transmission. Beside ongoing MDA program, results may be used equally in the development of group specific health awareness campaigns to educate and increase the consumption of DEC in the target groups of the endemic populations. It is also necessary to attempt changes such as, protection against mosquitoes using insecticide treated nets [43]. India is the leading LF endemic country in the world, the global elimination of LF depends much on the success of Indian continent. To achieve the goal of elimination of LF health officials, policy makers should make proper planning keeping in view the socioeconomic, environmental conditions and other logistics. Adhering to the above specifications filariasis can be eliminated from the India by 2020.

## Materials and Methods

### Study area

The study was undertaken in 30 villages from Karimnagar district of Andhra Pradesh from 2004 to 2007. These villages were marked as endemic zones by Andhra Pradesh state Government health authorities, where MDA programs have been undertaken since 2000. Karimnagar district lies on the Northern part of Andhra Pradesh approximately between the 18°25′48″N, 79°9′0″E. The occupation of the populace surveyed in the selected villages of the district varied for each individual; in some parts we encountered people who are full time agriculturist, or engaged as labourers in the agricultural activity. In most of the villages the populace was eking a living by working as labourers, or they were rolling the tobacco leaf for making beedis (rural form of cigarette in India), weavers and also people who were into small time business. The topography of the district is generally undulating and the altitude varied between the lowest (117 mt) and the highest (431 mt) in the villages where the study was done.

### Study design and socioeconomic data collection

Before commencing investigations, the local authorities and the residents of the selected villages were informed about the proposed study and obtained their written consent. The respondents/participants were selected by stratified random sampling methodology from all parts of the village. During the survey epidemiological (to asses the microfilaria (MF) infection), entomological and socioeconomic data were collected simultaneously by involving two sets of health volunteers. The socioeconomic details were collected only from people who were subjected to epidemiological study. Information on family characteristics with a possible influence on filariasis like sex, age, use of mosquito avoidance measures (like bed net, coils, any other or no protection measures), awareness on filariasis, number of children in a family, place of residence, family's monthly income, house structure (living in a hut, thatched, tiled and reinforced cement concrete (RCC) structure), education details, occupation information, vector breeding habitats, whether they participated in mass drug administration (MDA) program etc., was collected through interviewing the head of the family and other family members using a structured questionnaire in English or in the local language, Telugu. The questionnaire was composed according to local requirements and appropriateness.

### Parasitological test

Using finger prick method, 20 µl of blood sample was collected from randomly selected 40 house holds per village (five persons from each house hold) between 20.00 h and 23.00 h. A total of 200, blood smears (40×5 = 200) was collected and stained with JSB-II (Jaswant-Singh-Bhattacherji) stain and then checked under microscope for microfilaria (MF) of *Wuchereria bancrofti*.

### Ethics Statement

The study received ethical clearance from the Ethical Committee which was constituted in our institute (Indian Institute of Chemical Technology) affiliated to Ministry of Science and Technology, Govt of India. This ethical committee has approved to carry out the research work. The consent of the subjects who provided the blood sample was obtained as written consent before the commencement of epidemiological survey. All participants in the survey/questionnaire element of the study was also provided as written consent.

### Measurement of the socioeconomic variation

To obtain a measure of the socioeconomic status, proxy measures for economic well-being, like occupation, age groups, education details, monthly income, house structures, drainage system, mosquito breeding habitats and participation in MDA program were collected from the individuals and used in this study. Information on such asset variables was used to generate eigenvectors (weights) by Principal Components Analysis (PCA) [44] using a correlation matrix: the higher the eigenvector of a variable, the stronger its association with a high socioeconomic status. Assets that are unequally available to households have higher weights in the PCA. Missing values of distinct binary asset variables were replaced by the means of all summarized ‘0’ values (asset not present) and ‘1’ values (asset present) of this variable.

### Statistical analysis

SPSS version 15.0 was used for statistical analysis. Frequency distribution of different socio economic variables was calculated and occurrence of filariasis was compared with these variables by chi square. Socio economic index was derived by PCA. Risk estimates (Odds ratio) for different variables with filaria were calculated using bivariate logistic regression. Odds ratio with 95% CI were calculated for all independent variables (socioeconomic factors) and filariasis prevalence as dependent variable using multivariate logistic regression with forward stepwise method. Level of significance was considered as 0.05.
